# Scale Issues in Remote Sensing: A Review on Analysis, Processing and Modeling

**DOI:** 10.3390/s90301768

**Published:** 2009-03-13

**Authors:** Hua Wu, Zhao-Liang Li

**Affiliations:** 1 State Key Lab of Resources and Environmental Information System, Institute of Geographic Sciences and Natural Resources Research, Chinese Academy of Sciences, Beijing, 100101, China; E-Mail: wuh.06b@igsnrr.ac.cn; 2 Graduate University of Chinese Academy of Sciences, Beijing, 100049, China; 3 TRIO/LSIIT(UMR7005 CNRS)/ENSPS, Bld Sebastien Brant, BP10413, 67412 Illkirch, France; E-Mail: lizl@igsnrr.ac.cn

**Keywords:** Scale effects, Scaling, Scale domain, Scale threshold, Remote Sensing

## Abstract

With the development of quantitative remote sensing, scale issues have attracted more and more the attention of scientists. Research is now suffering from a severe scale discrepancy between data sources and the models used. Consequently, both data interpretation and model application become difficult due to these scale issues. Therefore, effectively scaling remotely sensed information at different scales has already become one of the most important research focuses of remote sensing. The aim of this paper is to demonstrate scale issues from the points of view of analysis, processing and modeling and to provide technical assistance when facing scale issues in remote sensing. The definition of scale and relevant terminologies are given in the first part of this paper. Then, the main causes of scale effects and the scaling effects on measurements, retrieval models and products are reviewed and discussed. Ways to describe the scale threshold and scale domain are briefly discussed. Finally, the general scaling methods, in particular up-scaling methods, are compared and summarized in detail.

## 1. Introduction

The advance of remote sensing technology in the 20th century has provided a powerful means to conduct regional and global measurements. Remote sensing technology can quickly access a wide range of real-time land surface spatial information and provides an effective way for resource surveys, environmental monitoring and disaster prediction. With the help of remote sensing technology, one can get geo-information quickly, accurately, efficiently and comprehensively. Undoubtedly, remote sensing will play an increasingly important role in the field of geosciences.

At present, remote sensing technology has entered an era of quantitative analysis. Thus, the important issues – scale effects and scaling – have already become one of the most important research focuses of remote sensing [1,2]. The scale represents the window of perception [2], the ability of observation, and reflects the limitation of knowledge through which a phenomenon may be viewed or perceived [1]. Consequently, scale issues should be carefully dealt with in remote sensing [3]. On the one hand, most retrieval models and algorithms are basically derived at small scales, implying that the land surface is homogeneous. If the models and algorithms are used at large scales, they may produce certain errors [4]. On the other hand, geo-information which is closely related to human cognition has the concept of regional scale, for example, weather forecasting, environmental monitoring, crop growth and yield estimation, disaster assessment, resource survey and so on. In short, the description of a process must be expressed at a certain scale. For instance, soil moisture content that is estimated from a small piece of farmland cannot be used as a drought index for large-scale agricultural drought monitoring. In other words, small scale information cannot be used as a substitute for regional scale information. The discrepancy between observation scale, model scale and land surface process scale may lead to different conclusions in the processes of monitoring and forecasting. As a result, the scaling of geo-information is inevitable for many disciplines. Scale effects constrain the accuracy of retrieval and limit the development of remote sensing applications. Consequently, they are becoming emerging problems which attract more and more the attention of scientists. Firstly, in order to analyze the scale issues of remote sensing, the problems of scale and the importance of scale will be reviewed and discussed.

As we know, research about scale has already permeated various disciplines, such as hydrology [5–7], meteorology [8], ecology [9,10] and geography [1,2,11]. The reasons why we need to transfer information across scales are simple. The instrument is one of the reasons; different instruments have different instantaneous fields of view that correspond to different spatial resolutions. In addition, the scale at which the available data has been measured is usually different from the scale required by the models. Again, the scale at which these models operate often varies from model to model. Furthermore, several factors, such as manpower, finances, time and other resources, constrain the choice of scale. For example, running a general circulation model will take a long time, even with a high-performance computer. Under the limitation of resources, a larger scale may be preferable. Finally, the scale of model output may not be in coincidence with the policy scale at which the decision is made. Taking into account these factors, the reason why more attention has been paid to scale issues, is easy to be understood.

Obviously, research is suffering from a severe scale discrepancy between data sources and the models used. Both data interpretation and model application become difficult due to scale issues. Openshaw [12] proposed the Modifiable Areal Unit Problem (MAUP), which means the results of studies could be different using different areal units and is thought of as the combined output of a scale problem and an aggregation problem. That is to say, different spatial processes may operate at different scales, and thus conclusions based on one scale may not be applicable to another scale. Again, spatial patterns are also scale dependent. Spatial patterns may look like a cluster at one scale but random at another [13]. Therefore, remote sensing can be thought of as a particular case of the MAUP [2]. Model application should be carefully dealt with rather than data interpretation. Retrieval models used in remote sensing are usually developed at a local scale, implying that models are merely suitable when the medium where the process takes place is homogeneous [4]. If these models are directly applied to a large scale, scale effects may be generated. In addition, the dominant factors are not the same at different scales, and they may lead to different retrieval models for different scales. Depending upon the observation scale, the process which appears homogeneous at a local scale may become heterogeneous at a large scale and parameters and factors which are important at one scale may become trivial at another [3,14,15]. For example, in the research on heat-water exchange, the turbulence flux that can be ignored at the large scale has to be taken into account at the local scale. Furthermore, the action of coupling different types of models makes the problem more severe. Atmospheric processes often occur at larger spatial scales and smaller temporal scales than hydrological processes [5,16]. How to couple these two types of models is not a simple question to answer. Without thorough consideration, using data and models at an inappropriate scale could lead to a meaningless conclusion [13].

To scientists, scale is undoubtedly one of the most important bases of research. What we study cannot be disengaged from the scale. Goodchild and Quattrochi [1] pointed out that scale is important because it not only defines the limits of our observations of Earth, but also is often a parameter in the physical and social processes which shape geographic phenomena. Scale research is also an important step in the campaign of validation of remote sensing products [17]. As spatial resolution becomes coarser, a scaling method must be developed in order to determine the accuracy of retrieval products [18]. Furthermore, the scale may determine the reliability of the research. From microscopic and macroscopic scales, we can understand both details and trends. It is helpful to thoroughly interpret geographic phenomena. In addition, the scale may determine the cost of research because detailed data is generally more expensive to measure, process and analyze. Undoubtedly, the development of scale research can relate the data measured at different scales and make use of this data effectively. It will solve the problems discussed above and drive the evolution of other relevant disciplines.

The aim of this paper is to demonstrate scale issues from the points of view of analysis, processing and modeling and to provide technical assistance when facing scale issues in remote sensing. The next sections will be organized as follows. In Section 2, some basic definitions will be given, as researchers may not seem to have agreed on the meaning of such concepts. They are the basis of analysis the scale issues. In Section 3, the mechanism of scale effects, which may benefit the scaling model, is briefly discussed. The main causes of scale effects and the scaling effects of the measurements, retrieval models and products will be reviewed and discussed. These are the key points in resolving scale issues in remote sensing. If the scale effects could be estimated correctly, the scaling model would become easier. One of the great advantages of remote sensing is its capacity for providing data at multi-scales, which is increasingly used to evaluate the influence of scale effects for identifying structure or patterns and modeling results. This raises a problem: where is the interval in which the phenomenon or the structures are nearly invariable or slowly variable? And, what is the validation scope of the retrieval models? In Section 4, the quantitative descriptions of scale threshold (invariance of scale) and scale domain are given. The analysis of scale threshold and the scale domain is imperative for understanding the dynamics of landscapes. In addition, they can be thought of as the linkage point between heterogeneity and homogeneity, which is closely related to the scale effects. Demonstrated in Section 5 are a few general scaling approaches, especially the up-scaling method, to characterize the influence of scale. Although the hypotheses and starting points are not the same, all roads lead to the same end result. These scaling methods can enhance upscaling from one scale to another. Under different circumstances, however, we can choose different methods.

## 2. The definition of scale and relevant terminologies

### 2.1. Notions of scales

In the field of scientific research, scale is often one of most involved terminologies. The scale can refer both to the magnitude of a study (e.g., its geographic extent) and also to the degree of detail (e.g., its level of geographic resolution) [1]. There are several terms that share the same meaning with observation scale that describe the detail, such as resolution, grain, support and so on. With remote sensing, scale might be resolution and can be thought of as the smallest objects being distinguished by sensors. For ecology, scale is likely to be grain, which is the measured size of patches. To environmental research, scale could be support, the largest area or time interval in which the parameter of interest is homogeneous [15]. To cartography, scale may be defined simply as the ratio between distance on the map and on the ground. There is much confusion and abuse concerning the term “scale” and it is necessary to identify the meaning of this word. Lam and Quattrochi [13] and Cao *et al*. [3] pointed out that at least four meanings of scale can be identified within the spatial domain. They are the observation scale, the operational scale, the geographic scale and the cartographic scale. Bloschl and Sivapalan [5] and Bierkens *et al*. [15] proposed another two meanings of scale: the modeling scale and the policy scale, respectively. From analysis, modeling and demonstrating perspective, scale needs to be divided into at least such six types. Here are the definitions used by the authors [3,5,13,15] with some modifications combined. Table 1 shows a comparison of the six meanings of scale used in various fields of scientific research.

1. Observation scale can be called a “measurement scale”. It depends on the method or the characteristics of the instrument and can be thought of as measurement units (i.e., intervals or areas or volumes) at which data is measured or sampled. To remote sensing, the measurement scale refers to the description of resolution, time interval, spectral range, solid angle or polarization direction. As the limitation of data collection and storage capacity, the smaller measurement scale usually corresponds to the smaller geographic scale and vice versa.
2. Modeling scale is the scale at which the model is built or derived in order to give reliable output. Both the measurement scale and the operational scale may influence the modeling scale. Observations sampled at a measurement scale are used as input for models, so the measurement scale must coincide with the modeling scale. If the measurement scale is smaller or larger than the modeling scale, it should be scaled. Again, a model needs to reveal the process; the modeling scale should also coincide with the operational scale. Similarly, it also needs to be scaled.
3. Operational scale refers to the scale at which a certain process is supposed to operate. It can also be called the “scale of action”. For example, thunderstorms may happen in an area of dozens of square kilometers. The operational scale of thunderstorms may be dozens of kilometers. It can be defined either as spatial extent (the lifetime), period (cycle) or the correlation length (integral scale), depending on the nature of the process [5]. Here, if the operational scale is smaller than the modeling scale, the variability lower than the modeling scale may be lost and the process may not be observed or found.
4. Geographic scale, which is also called “coverage”, refers to the spatial extent of research. It determines the biological organization level on which the surface property is observed, such as the leaves (a few centimeters), the canopy (10 to 100 m), the landscape (100 m to a few kilometers) or the region (about 100 km) [19]. A larger geographic scale study involves a larger spatial area, and a smaller geographic scale study only contains a smaller spatial area. The ratio between geographic scale and measurement scale often determines data volume and constrains storage and processing capacities.
5. Policy scale is the scale at which the decisions are made or the policy is implemented [15]. For example, whether the crop yield of one specific village is reduced or not, may be judged on village level on the basis of one year. In order to infer a reliable conclusion, the policy scale should be larger than the operational scale.
6. Cartographic scale is defined simply as the ratio between distance on the map and on the ground. It is often used to represent the spatial distribution of research results. Generally speaking, a smaller cartographic scale corresponds to a larger geographic scale and may show fewer instances of features or less detail when compared to a larger cartographic scale.

The six meanings of scale described above are mutually related. It is indispensable to determine the desirable scale before investigation. But, how do you select the suitable scale in remote sensing? Generally speaking, the choice of scale may only depend on the goals of the study if you do not take other factors (i.e., manpower, finance and time) into account. Commonly, the policy scale is determined first. And then, the operational scale is decided based on the previous knowledge of the research. Due to the fact that the policy scale is selected by the decision-making department and the operational scale is the natural characteristic of the process, they may have nothing to do with remote sensing. Remote sensing may only be used to provide knowledge to reveal the actual operational scale. By comparion, the observation scale, modeling scale and geographic scale are more closely related to remote sensing. They are more or less determined by the application of remote sensing. The smaller observation scale is not always correct. For example, the optimum observation scale for classification in land use and land cover is the scale where the variability within classes is at its minimum and the variability between classes is at its maximum. On the whole, the geographic scale should be large enough to characterize the image spatial variability or structures. At the same time, the observation scale and modeling scale should be smaller than the operational scale and be mutually consistent with each other. The result is not reliable when the observation scale and the modeling scale are totally different. Finally, the cartographic scale is determined to show the results and images which serve for decision-making after the research.

### 2.2. Characteristics of scales

Although “scale” is a widely used term and has different meanings in various disciplines, in general, it can be thought of as having multi-dimensionality, complexity and variability. Firstly, scale has a multi-dimensional nature [11]. It can be expressed as the variability in space and the expansibility in time [1]. Consequently, the scale is divided into spatial scale and temporal scale, respectively, based on the different research targets or fields. As the observations of remote sensing are more specialized compared to conventional measurement methods, the scale notion can be extended to spectral scale, directional scale and polarization scale. The spectral scale expresses the ability to discriminate fine spectral differences and refers to the full width at half maximum (FWHM) of band. The directional scale describes the angular geometry of the sun–object–sensor system [11]. The polarization scale refers to the polarization direction of the signal. In general, the spatial and temporal scales are closely related. Then, within a specific discipline, the processes that operate over larger temporal scales may also operate over larger spatial scales due to the transport mechanisms between space and time [1]. Bloschl and Sivapalan [5] demonstrated a scheme to classify hydrological processes according to typical space and time scales. Skoien *et al*. [20] used the ratio of spatial scale and temporal scale to discriminate the different processes in a space-time coordinate system. In reality, all sensors boarded on spacecrafts are a kind of “hand-made” equipment. The spatial resolution, the bandwidth and the spectral response function are not the same. The difference between sensors must be analyzed first. That is to say, both spatial and spectral scaling is necessary when comparing the retrieval products of different sensors. Even if the resolutions of the sensors are close to each other, i.e., MODIS and AVHRR, spectral scaling is needed. Such a point of view may improve our understanding of scale effects.

Secondly, scale has complex hierarchies. It is the reflection of the level of the organization of nature, which results in the research targets varying with scales. For example, small-scale hydrological studies may mainly focus on the scale of vegetation and soil; meso-scale studies may focus on the response of the hydrology unit to the changes of land surface; while large-scale studies may be mainly about the interaction of the atmosphere and the land surface. These phenomena and processes occurring at different scales may interact with each other, consequently, many regional or global changes, such as pollution, the greenhouse effect and biodiversity may be rooted in local scale or small-scale environmental problems. Similarly, large-scale changes (such as global climate change and ocean circulation anomalies), in turn, will influence small-scale phenomena and processes. This shows that both large-scale and small-scale studies are equally important. Large-scale describes the abstract features or macro-structure, and small-scale characterizes the details. On the one hand, we can understand the macro-changes and the general trend through large-scale studies; on the other hand, we may find the mechanism of the development of the process and give reasonable explanations through small-scale studies.

Finally, scale may also have variability, that is, the targets at different scales will show different characteristics. The isothermal surface would become non-isothermal. The spectral curve of emissivity may become smoother when the spectral scale is coarser. It has increased the difficulty of scale analysis.

### 2.3. Scale threshold and scale domain

With the development of scale research, scientists have found that the dominant factors which affect the processes change with the scale. Marceau and Hay [2] pointed out that the degree of explanation in the variation of normalized difference vegetation index (NDVI) varies with the scale. The variation of NDVI is mainly affected by the local scale topographic orientation when the resolution is finer, which shows the effect of solar radiation on vegetation in terrain areas. When resolution decreases, the elevation gradually becomes the dominant factor to describe the distribution of vegetation [2,14]. Based on these facts, scientists have proposed the concept of scale domain and scale threshold. The scale domain can be considered as the interval in which the phenomenon or the structures are nearly invariable or slowly variable, while they may change dramatically in a different scale domain that is separated by the scale threshold. The understanding of these concepts would benefit the analysis of the processes. In the same scale domain, scaling may be easier as the dominant factors of the processes are the same or similar. However, scaling may become more complex across scale domain due to different dominant factors. Therefore, one of the focuses of scale research is to determine the scale domain and the scale threshold [2].

### 2.4. Scaling and scale effects

Apart from the concept of scale, we have to pay attention to the terms, “scaling” and “scale effects”. Scaling, is just defined as transferring information across scales [16]. When one speaks of scaling, one must distinguish between two cases: up-scaling and down-scaling. Up-scaling is a process that transfers information from local scale to large scale. It concerns the extraction of global parameters from local measurements and has been much more studied [4]. On the contrary, down-scaling is to go from large scale to local scale. In general, up-scaling and down-scaling may also be called aggregation and disaggregation, respectively [18]. Scale effects refer to the contrast of information or the different characteristics at different scales. For example, the production of farmland estimated from TM and AVHRR are significantly different. The difference varies with the region and lacks regularity. It is mainly due to the dependence on the scale. How to select the appropriate scaling method and determine scale effects will be clearly analyzed next.

## 3. Mechanism analyses of scale effects

**Figure 1.**
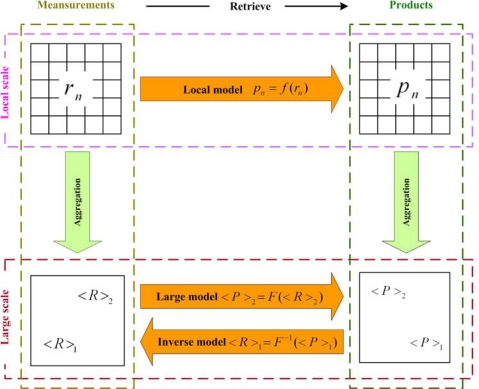
The relationship of measurements, retrieval model and products at different scales.

In order to analyze the mechanism of scale effects, firstly, we need to abstract the retrieval process from reality, which is concluded as follows:

(1) products=f(measurements)

where measurements refer to the physical quantity measured by remote sensing; *f* refers to the retrieval model that is used to estimate products from measurements; products are the characteristic parameters of land surface, such as biophysical (e.g., leaf area index, fraction of photosynthetically active radiation absorbed by vegetation) or geophysical variables (e.g., albedo, emissivity). The measurements, retrieval model and products may not be the same at different scales. So they can be considered as scale-dependent. The relationships are demonstrated in Figure 1.

Here, *r_n_* or < *R* >, *f* or *F* and *p_n_* or < *P* > represent the measurements, retrieval models and products at the local or large scale, respectively. Apparently, if the retrieval models at both local scale and large scale are available, there is no scale effect yet. The products retrieved by remote sensing can be estimated by the corresponding models. However, only retrieval models at a local scale are usually proposed, as they can be easily validated in the laboratory or the testing field. Generally, the retrieval model may not be the same for different scales as the dominant factors or stated variables are variable at different scales. For example, both Lowtran and Modtran are radiative transfer models, however, they are only suitable for the low spectral scale and the moderate spectral scale, respectively. In such a situation, the scaling for the retrieval model is necessary. The first task of scale research in remote sensing is to determine the validation scope of retrieval models. Based on physical analysis, the retrieval models are simplified or re-parameterized to adapt to the new scale. If we do not scale retrieval models and only adopt the same form at different scales, there are two other alternative methods to compensate for the scale effects: the scaling of measurements and the scaling of products. If we assume the retrieval models are the same at any scale, there are two ways to estimate measurements or retrieve products at a large scale. One is to aggregate measurements and products directly using local scale data, thereby producing average measurements < *R* >_2_ and distributed products < *P* >_1_. The other is to use < *R* >_2_ and < *P* >_1_ through the retrieval model and the inverse model to generate the corresponding ones, thereby producing lumped products < *P* >_2_ and equivalent measurements < *R* >_1_. It is difficult to determine which are best. We can only select the appropriate one by real situations. For example, the goal of scaling for leaf area index (LAI) is to make the values derived from coarse resolution sensor data equal to the arithmetic average of values derived independently from fine resolution sensor data [21]. If the retrieval model is proposed at local scale and the products estimated are associated with the unit area, such as LAI, < *P* >_1_ may be more suitable because it follows the law of conservation of matter. Otherwise, < *P* >_2_ may be more advisable. The product of temperature is an example. Here is the other thing we need to pay more attention to. The aggregation may not be area-weighted, the aggregation of radiance in a heterogeneous terrain region should consider both the area and the local slope angle effects [22]. Besides, not all the aggregation is scientifically reasonable. As the aggregation of temperature follows neither the law of conservation of energy nor the law of conservation of matter, consequently, it may not make sense. The discrepancy between < *R* >_1_ and < *R* >_2_, and < *P* >_1_ and < *P* >_2_ may be the focus of scale research. From the discussion above, the research on scale effects and scaling in remote sensing should begin around the points of view of measurements, retrieval models and products.

### 3.1. Main causes of scale effects

The main causes of scale effects can be summed up in three main reasons from the perspective of analysis, processing and modeling.

The first reason is the limitation of measurement. Any measurement equipment has its own scale representation. It can only reflect the specific information within the scope of observation. An infrared radiometer at ground level can merely represent the temperature at the scale of points; however, the Large Aperture Scintillometer (LAS) can reflect the exchange of energy at the scale of a region. Zhang *et al*. [23] have shown that the criterion for judging whether the surface temperature is isothermal or not may only be dependent on the spatial scale or the spectral scale through experimental verification. Along with the change of scale, the isothermal surface could become non-isothermal.

The second reason is the scale applicability of the retrieval models. The retrieval models do not explicitly express the characteristics of scale; however, they may be suitable for homogeneous surface or point measurements [4]. Chehbouni *et al*. [24] pointed out that it is not appropriate to use relationships between model and observational variables developed and calibrated at a local scale for application at a larger scale just by scaling the parameters. As a result, they need to be simplified or re-parameterized to adapt to the new circumstances since the driving force or mechanism may be totally different at various scales. It is difficult to imagine that models at the scale of leaves are still applicable to the scale of canopy. Consequently, the models or algorithms proposed at one scale would be neither effective, nor similar, or need to be revised at any other scale. Therefore, the first step of scale research is to investigate the impact of scale on the mechanism of physical models and algorithms. At present, the scale applicability of Lambert’s assumption [25], Beer’s law [26], Helmholtz’s reciprocity principle [27], and Planck’s Law [28,29] have already been discussed at the pixel scale in remote sensing applications. The results showed that we need to carefully consider the scale’s applicability for retrieval models when using them at different scales.

The third reason is the heterogeneity of land surface and the characteristics of linearity or nonlinearity of the retrieval models. These two factors affect the scale effects together. If the measurements are homogeneous, it would not cause scale effects no matter whether the retrieval models are linear or not. The heterogeneity could be thought of as the inherent nature of land surfaces, which are a mosaic of different cover types. In other words, heterogeneity would be considered as the surface properties vary over the observed scene [19]. When observing through sensors, it may be very heterogeneous, especially in the case of coarser spatial resolution sensors. As the resolution decreases, the possibility that one pixel contains more than one cover type would increase. Therefore, heterogeneity is the most fundamental characteristic of all landscapes [30]. If land surface measurements or media properties vary in observation unit (space), it can be thought of as heterogeneity [5]. The surface heterogeneity is often of great concern when deriving surface parameters using remotely sensed data [31]. Here, the heterogeneity may be caused either by the change of density or the discontinuity that means the contrast change between cover types. The scale effects due to density change are usually smaller and can be negligible [31, 32]. On the whole, the term heterogeneity is a relative concept. The homogeneous canopy may still cause the heterogeneity of temperature due to the shade. In order to limit the influence of heterogeneity on the description of land surface processes, Garrigues *et al*. [19] suggested two strategies. One is to quantify the intra-pixel spatial heterogeneity. The other is to define the proper pixel size to capture the variability of data and minimize the intra-pixel variability. Besides the heterogeneity of the land surface, the characteristics of linearity or nonlinearity of the retrieval models is the other factor. We cannot arbitrarily draw the conclusion that the linear retrieval models would not cause a scale effect or that the nonlinear retrieval models would cause a scale effect. The linear retrieval models could also incur scale effects when the retrieval models for different cover types are quite different [31]. The nonlinear retrieval models may also cause no scale effects when the medium is homogeneous, which has been successfully demonstrated by the Taylor series expansion [33,34]. Chen [31] suggested that nonlinear algorithms applied to pixels mixed with different land cover types may be the major cause of scaling errors. Considerable scale effects may be expected for mixed pixels where the mixture is unknown, as radiative signals from different cover types can be very different for the same measurements [32]. Generally speaking, the linear retrieval models may cause smaller scale effects than nonlinear ones in such a situation [31].

### 3.2. Effects of scale on the measurements, retrieval models and products

The mechanism analyses of scale issues undoubtedly involve several questions. The first one is what the main causes of scale issues are in remote sensing, which has already been analyzed above. The following one is what the effects of scale on the measurements, models and products are.

With measurements, what we are more concerned about may be the mean, variance and correlation lengths. Bloschl *et al*. [5] and Western *et al*. [35] have already demonstrated the effect of the measurement scale on the apparent variance and the apparent integral scale (apparent correlation length). Here, the “apparent” means the statistical properties that appear in the data. The apparent variance decreases with increasing resolution, while the apparent correlation length, the average distance (or time) over which a property is correlated, always increases with increasing resolution.

To the retrieval models, the effects of scale may be difficult to analyze. We may select suitable models at the corresponding scale; for example, the radiative transfer model of vegetation at the leaf scale [36] or canopy scale [37]. Whether the change of scale has an effect on the retrieval model is dependent on the status of the measurements and the form of the retrieval model. In the case of three-dimensional structures, the retrieval models may need to be remodeled. Smolander and Stenberg [38] used a clumping index to correct the radiation attenuation coefficient at a shoot scale and proposed the forest reflectance models for coniferous forests. In the case of big-leaf assumptions, if measurements are homogeneous, the scale has no effects on the retrieval model no matter if the model is linear or non-linear. If the measurements are heterogeneous, there is a scale effect on the retrieval model. There is only no effect when the retrieval model is linear and does not vary with the land cover types, which means the model has a uniform form. Otherwise, the retrieval model may need to be simplified or re-parameterized. Raffy [4] proposed a spatialisation model to overcome the scale effects by using the lower and upper bounds of the retrieval model. Tian *et al*. [21] addressed the problem of how the scale, or spatial resolution of reflectance measurements impacts retrievals of LAI and developed a physically radiative transfer formulation with explicit spatial resolution dependents.

To the products, the effects of scale have already been widely discussed. There is conflicting conclusions in the literature as to whether products are scale dependent or scale free. The main reason is that the scaling effects are usually dependent on the real application. If the retrieval model that is used is linear, there may be no scale effects, yet when the retrieval model has a uniform form for all the land covers, for example, if simply mapping the reflected solar radiation to the surface albedo, we can argue that the reflected solar radiation parameterization is scale invariant. Otherwise, the nonlinearity of albedo with topography and spectral dependence of albedo and the reflected solar radiation would be scale dependent. This demonstrates that a different parameterization and different assumptions related to the retrieval model can lead to different conclusions for the same physical process [39]. Raffy [4] used a convex hull to judge whether products are overestimated or not. The concave retrieval model overestimates the arithmetic average of values derived independently from fine resolution, while the convex retrieval model underestimates them. Garrigues *et al*. [34] pointed out that the magnitude of scaling effects increases rapidly with pixel size until the size is larger than the typical length scale of data for the univariate retrieval model. For the bivariate transfer function, the scaling effects are the combined effects of several components, which may add up or compensate for each other. The effects of scale on specific products are theoretically and practically analyzed. We may resort to relevant literature, such as bidirectional reflectance distribution function (BRDF) and albedo [40], the temperature [22], the emissivity [23], the infrared radiation and the reflected solar radiation from the surface [39], the latent, sensible and ground heat fluxes [16,39,41–44], carbon flux [45,46], soil moisture [47–50], NDVI and vegetation fraction [51,52], LAI [17,31,33,34,53,54], net primary production (NPP) and gross primary productivity (GPP) [55–57], directional gap fraction [58].

## 4. Quantitative descriptions of scale threshold and scale domain

After discussing on the main causes of scale issues and the effects of scale on the measurements, models and products, the analysis of scale threshold and scale domain becomes an another critical problem waiting to be resolved. It is the basis of understanding scale issues. At present, one great advantage of remote sensing is the capacity to provide data at various resolutions. It may become easier to identify the scale thresholds below by identifying which biophysical or geophysical variables are spatially dependent and whether they become less dependent or independent. Here, we may take the scale domain as the appropriate scale for a given geographical environment. The relevant knowledge of scale threshold and scale domain may benefit the understanding of the validation scope of the retrieval model, dynamics of landscapes. In the following, several representative methods will be presented. Although these approaches may not necessarily apply to all cases, they in fact provide effective ways to cope with the problems.

### 4.1. Geographic variance method (GVM)

Moellering and Tobler [59] proposed the geographic variance method to analyze the scaling effects of geographic phenomena. Geographic variance analysis, which is a hierarchical analysis, can determine the relative variability and independent contribution at each level in a nested hierarchy. Most spatial data can be constructed to a nested hierarchy by a simple aggregation approach, and then the geographic variance method can be applied [30]. According to this theory, the total variability can be divided using the sum of squares at each level, while most geographical phenomena may occur at the level where the level variability is the highest. In other words, the operational scale of a phenomenon coincides with the scale of maximum variability in the data. Wu *et al*. [30] argued that the geographic variance method may be a potentially powerful method to detect and describe multi-scale structures of landscapes. However, Cao and Lam [3] thought its validity remains unclear and more analysis is needed. In consequence, this method needs to be further investigated systematically in remote sensing.

### 4.2. Wavelet transform method (WTM)

The wavelet transform method, which is a relatively new mathematical technique, has already been widely used in various disciplines. It uses a localized function in time or space. The wavelet’s size can be adjusted and shifted to analyze a data set. Thus, we can investigate features of interest in the data set at an appropriate scale, for example, broad features at a large scale and fine features at a small scale. With the help of the wavelet transform method, we can find where changes in a data set take place and simultaneously measure how large these changes are. Percival [60] suggested that the wavelet variance calculated by the wavelet transform method is a natural tool to investigate the spatial scales of variability in remote sensing data. It can be used as an indicator to quantify the length scale of land surface. Through the analysis of simulating images, the hypothesis is confirmed that the dominant length scales in the landscape may correspond to the scale with the highest wavelet variance [16]. The best wavelet to identify length scales is the Haar wavelet. The results indicate that the wavelet transform method is also a suitable tool to analyze the heterogeneity of land surface and infer the optimum scale under which the main variability presented in the image may be lost. Apparently, GVM and WTM are different methods. One starts from the space domain, while the other starts from the frequency domain. However, these two methods will produce the same conclusion through several data simulations. It may be due to the fact that the cumulative variance and covariance are equal to the variance and covariance within the specific scale in the case of the Haar wavelet. Wavelet analysis would be a good tool to study multi-scale relationships of spatial pattern and heterogeneity [10]. However, the manner of WTM is also dependent on the mother wavelet. WTM’s potential for using other wavelets, such as Daubechies, Coiflet and so no, needs to be further explored.

### 4.3. Local variance method (LVM)

In order to choose an appropriate scale for a particular application, Woodcock and Strahler [61] proposed the local variance method, which is related to the relationship between the size of objects in a scene and the spatial resolution of sensors. The local variance uses the standard deviation as an indicator to reflect the mean value of the standard deviation of a moving window over the entire image. According to the graphs of local variance as a function of scale, the spatial structure of an image can be measured. The reasons are very simple. When the spatial resolution is considerably finer than the size of objects in the scene, most of the measurements in the image will be highly correlated with their neighbors and the local variance would be low. As the size of spatial resolution increases, it may approximate the size of objects, then the likelihood of neighbors being similar decreases and the values tend to be different from each other. It thus causes the local variance to increase. If the size of spatial resolution increases further, it would be greater than the size of the object, and the possibility of one single pixel containing many objects increases, then the local variance gradually starts decreasing. Therefore, the peak would appear when the size of spatial resolution matches the size of the objects. Multiple high local variance peaks may show that the scene has multiple scales of variation. For example, the local variance as a function of resolution for agricultural areas, may indicate two distinct scales of high variance, one related to the size of individual crop rows, and the other related to the size of the field [61]. With the help of analysis of local variance, the scale where the geographic phenomenon may occur can be found, and then the observational scale of study can be determined. Here, the local variance looks like the texture analysis in digital image processing [3]. The only difference is that the texture analysis method can use several indices, such as moments, min-max, entropy, and so on. The local variance method tries to find the “scale of action”, however, there are certain limitations involved in the usage, which are acknowledged by the authors. One limitation is that it is unrealistic to assume an idealized square wave on the part of the sensor and the pixel value of a coarse resolution image is simply an average of finer resolution pixels within corresponding coarse pixels. The other limitation is that it is dependent on the global variance in the image and the values of local variance cannot be directly compared between different images. Therefore, the relevant improvements should be made around these limitations.

### 4.4. Semivariogram based method (SVM)

The semivariogram is often used as a tool to measure the difference in property values at two sample locations as a function of the distance between these locations. It provides the mean characteristics of spatial heterogeneity at the image scale. There are three features to characterize the semivariogram: nugget, sill and range. These features can be used to characterize and quantify the spatial heterogeneity of a land surface [19]. The nugget is the discontinuity of the semivariogram at the origin. It can be used to judge if uncorrelated noise (measurement error) exists or spatial structures are smaller than the pixel size. The sill is the value that the semivariogram may reach when the distance heads toward infinity. It can be an indicator of the spatial variability of the data. The range is the distance at which it reaches a sill. It can be used to characterize the image spatial structures. Artan *et al*. [62] used the semivariogram and the characteristic length calculated from the spatial autocorrelation to determine the scale of variability of the remotely sensed data. In order to account for the multiple length scales of data, Wackernagel [63] and Garrigues *et al*. [19] proposed a linear combination of elementary variogram models to model the semivariogram. Then, the structural parameters of the semivariogram model can be generalized into a single parameter: the integral range (*A*). Here, its square root *D_c_* is a weighted average of several range parameters and quantifies the mean length scale of data. Based on Shannon’s theorem, the spatial sampling frequency must be larger than 2/*D_c_*, therefore, the pixel size must be smaller than *D_c_*/2 to retain the major part of spatial variability. Here, either *A* or *D_c_* can be used to judge whether the geographical scale is large enough to detect the length scales of the landscape. In addition, the rate of data regularization can be used to characterize the rate of the loss of image spatial variability at a given spatial resolution. Tarnavsky *et al*. [52] applied variogram modeling to evaluate the difference in spatial variability at different scales and characterize the impact of scale. Based on the analysis, an approach for selecting the spatial resolution is proposed. Subsequently, Garrigues *et al*. [64] proposed to use multivariate, red and near infrared spectral properties to quantify the landscape spatial heterogeneity by direct and cross-variograms modeled together with the geostatistical linear model of co-regionalization. The result showed it to be more powerful than univaritate variogram modeling.

### 4.5. Fractals method (FM)

The term “fractals”, first proposed by Mandelbrot [65], is now widely used in different science domains, such as biology, physics, chemistry, geography and so on. The reason why fractals attract more and more attention is that the real world is too regular to be measured or simulated by traditional methods [13]. Many curves or surfaces in the world may be statistically made up of copies of itself at a reduced scale. This statistical self-similar property is the key point to understanding the concept of fractals. In classical geometry, the dimension of a point is zero, a line is 1, a plane is 2, and a cube is 3. While, in fractal geometry, the fractal dimension D of an object can be any non-integer dimension [66]. For example, the fractal dimension of a curve can be any value between 1 and 2. The fractal dimension of a surface can be any value between 2 and 3. Empirical studies indicate that true fractals with strict self-similarity do not exist. However, the information that fractal dimensions provide with scale can be used to indicate the optimum measurement scale. There are many ways to determine fractal dimensions. Xia and Clarke [67] introduced several frequently used definitions of fractal dimension, which could be used to find the process scale. The turning points of fractal dimension may contain some important information. For example, they should be those where new patterns may emerge or resolutions approach dominant operational scales. Generally speaking, the more irregular an object, the bigger the fractal dimension. The fractal dimension of an image is expected to be lower as the resolution becomes coarser due to coarser resolution corresponding to lower variability and vice versa. Therefore, the scale at which the highest fractal dimension is measured may be the scale at which most processes operate [13]. Although no agreement has been reached on the definition of fractal dimension, it is a promising research direction. Table 2 summarizes the advantages and disadvantages of the above mentioned methods.

## 5. Overview of general scaling methods

In order to solve scaling problems and compensate for scaling effects, several authors have already developed some frameworks. These frameworks provide a few systematic approaches to characterize the influence of scale on the measurements, retrieval models and products. In order to better understand these approaches, we classify them into three main categories which will be briefly summarized below.

### 5.1. Scaling methods for measurements

Scaling methods for measurements are easier to deal with because the measurements recorded by remote sensors usually capture the radiance emitted or reflected by the surface. In such cases, the Area-Weighted Scaling Methods (AWM) may be applicable in a flat region. Otherwise, the influence of slope angle should be taken into account [22]. These methods were all developed on the basis of the law of conservation of energy or matter. If the retrieval models at both local and large scale are known in priori, the scaling methods mentioned above would work well. However, if the retrieval model at one scale, either local scale or large scale is known, the retrieval model derived from one scale cannot be guaranteed to be used at the other scale. Therefore, AWM alone is insufficient; correction items to the area-weighted measurements need to be added in order to scale products correctly. This process could be thought of as finding the representative measurements corresponding to a large scale. The aim of these methods is to get the scale invariant result at large scale when the local scale retrieval model is used. Bierkens *et al*. [15] proposed a scaling method, Finding Representative Parameters Method (FRPM), and gave an example of finding the representative conductivity for blocks in a numerical model of groundwater flow. Through the comparison of model output at both local scale and large scale, the representative conductivity can be thought of as the sum of two items. One is the arithmetic block average of parameters. Another is the block covariance of parameters. Through numerical analysis and comparison, we may give the analytical solution to the representative measurements in remote sensing. Then, the correction items may be the combined effects of the nonlinearity of the retrieval model and the heterogeneity of measurements.

### 5.2. The scaling methods for retrieval models

Generally speaking, we may only know the retrieval model at a specific scale. That is to say, we need to scale the retrieval models to the other scales through the appropriate assumptions and simplifications in order to not only consider the scaling effects but also provide scale invariant algorithms. Raffy [4] proposed a general method, the Computational Geometry Method (CGM), to reduce the scaling effects introduced by the heterogeneity of measurements and the nonlinearity of a retrieval model at a local scale. The method takes advantage of the convex hull of computational geometry to determine the interval where the distributed result may exist. Considering all possible distributions of measurements, the distributed result always falls into the interval of the lower and upper bounds of the retrieval model which measures the maximum error due to the scaling. Here, the bounding functions can be interpreted as a measure for the non-linearity of the model [16]. Subsequently, Raffy and Gregoire [68] used such a concept combined with a least square method to determine the coefficients of the semi-empirical model that is validated at a small scale and upscale the model to the large scale with global radiances. If there is not more information available other than the domains of measurements and the retrieval model at the local scale, one can expect to reduce the scaling effects by employing the model spatialization method, which is the function of the convex and concave function of the retrieval model at a local scale. Garrigues *et al*. [34] argued that the assumption that the retrieval model follows a uniform distribution in the interval between the convex and concave function is inappropriate within a moderate resolution pixel. This may be one of the reasons limiting the application of the spatialization model. For the specific retrieval model, a more specific scaling method would be used, the Physical Scaling Method (PSM) based on the radiative transfer theory. Tian *et al*. [21] developed this method with an explicit spatial resolution dependent in order to upscale LAI retrieved from AVHRR data to the coarser resolutions. Malenovsky *et al*. [11] discussed PSM and gave examples of the radiative transfer scaling in a Norway spruce forest stand.

### 5.3. The scaling methods for products

Compared to the scaling methods of measurements and retrieval models, the scaling methods for products are more widely studied in research. These scaling methods provide a few systematic approaches to characterize the land surface heterogeneity and compensate for scaling effects. Here, we just emphasize the spatial domain. Other scaling methods applied in different domains can be realized by certain approaches. For example, scaling in the temporal domain may consider the general diurnal patterns of meteorological variables [69] or assume that the evaporative fraction (EF) is constant throughout the day [70]. Scaling in the spectral domain and the directional domain would rely on experimental regression or look-up tables (LUT) to solve the scale problem, respectively. Since land surface is very heterogeneous, up-scaling is probably more important than downscaling in product validation. Therefore, we may be more concerned about the up-scaling method. The downscaling method resorts to relevant literature [18]. In general, the simple up-scaling method can use statistical algorithms to estimate the spatial means at a large scale by either area-weighted products of homogeneous patches or the integration over the probability density function of products [42]. In the following, other extensively used scaling methods in the spatial domain, such as Empirical Regression Method (ERM), Taylor Series Expansion Method (TSEM), Contextural Parameters Method (CPM), Statistical Fractal and Self-similar Method (SFSM) will be analyzed in detail.

ERM is simply used to empirically calibrate the relationship of products between fine and coarse scales [71–73]. The goal is to relate the products at different scales by regression. Fernandes *et al*. [53] simply used empirical relationships between spectral vegetation indices and surface estimations of LAI, which are in-situ measurements from the auxiliary sites, to estimate coarse-scale LAI. Martinez *et al*. [74] used a multivariate ordinary least squares (OLS) algorithm which uses an iteratively re-weighted least squares (IRLS) algorithm to build an empirical relationship and upscale the field LAI data to the corresponding satellite products. Although the scaling method is simple, the relationship of regression is usually site, time, model and scale dependent. If any one factor is changed, the relationship may need to be recalibrated. Consequently, it would gradually be replaced by other scaling methods.

TSEM is based on Taylor’s theorem of linearizing the retrieval model around the arithmetic average of measurements [16,33,34]. It characterizes the scaling effects as the combined effects of degree of non-linearity of the model and the heterogeneity of the land surface. The key to using this method is to know how to estimate the variance and covariance within the pixel at coarse resolution. Hu and Islam [33] proposed a novel method based only on the mean values of measurements at pixel scale to parameterize the variance and covariance terms by the half ellipse relationship between the normalized standard deviation of radiance and the normalized average radiance over the pixel. They used this relationship to study the effects of subgrid scale heterogeneity of soil wetness and temperature on grid-scale evaporation [75]. Garrigues *et al*. [34] used the local dispersion variance and covariance which are calculated by semivariogram and cross semivariogram from concurrent high spatial resolution images or a spatial sampling per type of landscape of the high spatial resolution data to quantify the spatial variability within the moderate resolution pixel. Afterwards, Garrigues *et al*. [76] proposed a spatio-temporal model of the variogram to estimate the intra-pixel spatial heterogeneity. It is a novel approach to predict the variogram at a date at which the high spatial resolution scene is not available by the seasonal cycle of phenological variability. Although its validity is argued by Chen [31], it is still useful for product scaling. In conclusion, the method is easy to understand and operate. If the intra-pixel spatial heterogeneity can be successfully represented, it can give reasonable results. The disadvantage of TSEM is that it must satisfy some hypotheses. The model needs to be continuous and have at least up to second order continuous derivatives in the interval under consideration. If the model has strong non-linearity, the approximation would not be appropriate. When using complicated models with a large number of variables, it would be difficult to find the derivatives of the model [16]. Again, failure to include high order terms or interactive terms (covariance) may limit the application of this technique.

Generally, the variance and covariance within one pixel are important for the traditional textural parameters that can capture the spatial variability of the surface. However, they are difficult to be estimated due to the fact that the concurrencies of high and low resolution images are often not available. In addition, they may not be used to discriminate the situations where the surface heterogeneity can be caused either by the cover type changes or by density change within the same cover type. If the surface heterogeneity is caused by cover type changes, the linear models which have different forms for different cover types may also cause scale effects. In contrast, if the surface heterogeneity is caused only by density change, the non-linearity of the model would generally cause very small scale effects [31, 32]. In short, TSEM would be useless when the model is discontinuous or piecewise against the measurements. In order to break through the limitations of using textural parameters, Chen [31] proposed a different scheme, CPM. It took the contextural parameters (e.g., the fractions of subcomponents) as a bridge to quantify the scaling effects. Simic *et al*. [55] tried to use such subpixel information to upscale NPP. The result showed that the correlation between the distributed NPP and lumped NPP was greatly improved. El Maayar and Chen [44] thought the scale effects resulted from the overlooking of sub-pixel variability of land surface characteristics and proposed a simple algorithm that used contextural parameters of vegetation, soil cover, and surface topography to correct evapotranspiration (ET) estimates. Jin *et al*. [54] developed such algorithms to remove the biases in lumped LAI maps using sub-pixel land cover-type information and correct coarse resolution products of LAI. Here the fractions can be estimated either by the sub-pixel land cover masks at high resolutions or the linear unmixing method. Braswell *et al*. [77] used the Bayesian-regularized artificial neural network with combined MODIS-MISR data to estimate sub-pixel land cover fractions and yielded a quantitative improvement result over spectral linear unmixing of single-angle, multispectral data. The result is promising. However, contexture-based methods are usually model-dependent. They cannot provide a general method to compensate for scaling effects. The mapping function needs to be redefined when the model is changed. When the model has several variables, the mapping functions become very hard to be derived. Therefore, contexture-based methods are not alternatives to texture-based methods. They are new attempts to solve scaling effects. In practice, we need to choose the corresponding scaling methods according to actual requirements.

SFSM is based on the simple scaling and multiscaling characteristics of products. Dubayah *et al*. [78] have found the log-log linearity relationship between the statistical moment and the scale factor, and the non-linear dependence of scaling exponents with order moment. That is to say, the statistical properties (moments) of process can be extended from one scale to the other scale. Hu *et al*. [47] analyzed the statistical characterization of soil moisture retrieved from remotely sensed passive microwaves. They found these properties and suggested a deviation from simple scaling and the possible presence of multiscaling [49,79]. Das and Mohanty [50] argued that the scaling exponent of soil moisture during dry-down suggests a transition from simple scaling (in wet fields) to multiscaling (in dry fields) behavior. However, the fluctuation parts of soil moisture, which is decomposed by wavelet transforms, showed a simple scaling characteristic which implied the possibility of scaling soil moisture. In addition to soil moisture, other products also behave with self-similarity characteristics, such as NDVI and radiometric temperature [80]. Based on the multiple moments scaling law, the wish of scaling between different scales without other prior knowledge may be realized.

Table 3 summarizes the general scaling methods discussed above. Besides these methods, we can use other methods for scaling the products, such as disaggregation-aggregation combined methods[81], process simulation methods [9] and so on. Again, additional information provided by optical and thermal sensors can be used as auxiliary knowledge to assist the scaling. Kustas [82] utilized such information and proposed the DisTrad technique to estimate subpixel variation of surface temperature. On the whole, scaling methods for products have already become a universally recognized problem. Its development needs the development of cross-disciplines in order to achieve theoretical and technical innovation.

## 6. Conclusions

Scale has already been recognized as a crucial concept in the description of the hierarchical structure of our world. Undoubtedly, remote sensing will advance the development of scale research. In reviewing the scale issues in remote sensing from analysis, modeling and demonstrating perspective, we found that there is no universal scaling method. Each has specific problems and limitations, although they could provide the possibility for solving scaling problems and compensating for scaling effects. The decision as to which method to use depends on real situations. The reason why no one method is proved to be effective may mainly stem from the heterogeneity of land surface and the nonlinearity of the retrieval models. We may expect the advancement in the understanding of the scale threshold and scale domain would bring rapid progress of scale research in remote sensing.

Although important steps have already been made, the research concerning scale is still in the initial stage and the scaling methods are not mature. In the future, we may pay more attention to: 1) How to effectively scale data with the concurrence of both local and large scale data. The optimal scale to estimate land surface parameters, the relationship between scale domain and coefficients of scaling can be deeply investigated when the data at different scales is available. 2) How to characterize the inner pixel heterogeneity without the concurrence of local data. We may use other spectral bands (i.e. optical, thermal or microwave sensors) or combine the Land Model with time-series data to estimate the heterogeneity within the pixel. 3) How to well combine the TSEM and CPM and absorb the advantages of both methods. The effect of heterogeneity caused either by the change of density or the discontinuity on scaling can be resolved. 4) How to well define the input variables, models and output parameters. Different parameterizations may result in different conclusions. Furthermore, the clear separation of system errors from the retrieval model and scale effects is also the key perspective of scale research. 5) How to effectively validate products at different scales. This needs the development of scale research. We will face the challenges to bridge the gap between theory and application.

## Figures and Tables

**Table 1.** Comparison of the six meanings of scale used in the field of scientific research.

**Meaning**	**Description**	**Remarks**
Observation scale	The measurement units at which data is measured or sampled	Referring to the description of resolution, time interval, spectral range, solid angle or polarization direction.
Modeling scale	The scale at which the model is built or derived	In order to better reveal the process, the modeling scale should be coincided with both the observation scale and the operational scale.
Operational scale	The scale of action at which a certain process is supposed to operate.	Depending on the nature of the process. Variability lower than modeling scale may be lost if the operational scale is smaller than the modeling scale.
Geographic scale	The spatial extent of research	A larger geographic scale study involves a larger spatial area and a smaller geographic scale study only contains a smaller spatial area.
Policy scale	The scale at which the decisions are made or the policy is implemented	In order to infer a reliable conclusion, the policy scale should be larger than the operational scale.
Cartographic scale	The ratio between distance on the map and on the ground	A smaller cartographic scale corresponds to a larger geographic scale and may show fewer instances of features or less detail.

**Table 2.** Comparison of different methods used to quantitatively describe scale threshold and scale domain.

**Methods**	**Advantages**	**Disadvantages**	**References**
GVM	- A hierarchical analysis to determine the relative variability and independent contribution at each level. - The data set can be divided into any arbitrary nested scale.	Its validity remains unclear and more analyses are needed.	[3] [30] [59]
WTM	It can investigate features of interest in the data set at an appropriate scale and find the length scale of the variability.	- The dimension of data set must be the exponent of 2. - The manner of WTM is dependent on the mother wavelet.	[16] [60]
LVM	The principle is easy to be understood.	- It is unrealistic to assume the pixel value of a coarse resolution image is simply the average of finer resolution pixels within the corresponding coarse pixel. - It is dependent on the global variance in the image and the values of local variance cannot be directly compared between different images	[61]
SVM	- It can be used to judge whether the geographical scale is large enough to detect the length scales of the landscape. - The loss of image spatial variability at a given spatial resolution can be estimated.	The second order stationarity hypothesis should be satisfied.	[19] [63]
FM	- It has a theoretical basis that many curves or surfaces in the world may show the statistical self-similar property. - The more irregular an object, the bigger the fractal dimension. The turning points of fractal dimension may contain some important information.	No agreement has been reached on the definition of fractal dimension which can be used to determine the characteristic scale.	[13] [67]

**Table 3.** Summaries of general scaling methods used in remote sensing.

**Categories**	**Methods**	**Advantages**	**Disadvantages**	**Ref.**
Scaling methods for measurements	AWM	- Simple principle. - Easy usage.	- May be only suitable for flat regions.	[22]
	FRPM	- Simple principle. - The analytical solution to scale measurements.	- The representative parameters may have no specific physical meanings. - It is difficult to get representative parameters when facing a large number of input arguments.	[15]
Scaling methods for retrieval models	CGM	- Regardless of whether or not retrieval models are continuous or derivable.	- Does not take into account the actual distribution of parameters. The weights for lower and upper bounds of a retrieval model may be inappropriate. - Needs a large amount of computing time and a special algorithm to retrieve convex hull, when facing a large number of input arguments.	[4] [16] [68]
	PSM	- More accurate.	- It is difficult to derive when facing a large number of input arguments.	[11] [21]
Scaling methods for products	ERM	- Simple principle. - Easy usage.	- Less accurate.	[53] [74]
	TSEM	- Better basis of mathematics. - Easy usage.	- The retrieval model and its derivatives must be continuous in the domain. - It may cause greater error when the model is strongly non-linear. - The model needs to use the local variance as input, which may usually not be available.	[16] [33] [34]
	CPM	- Taking the discontinuity as the main cause of scale effects. - Easy usage.	- Neglects the heterogeneity within certain land types. - Has no theoretical or physical basis.	[31] [55] [44] [54]
	SFSM	- Simple principle. - Grasps the simple scaling and multi-scaling characteristics of surface nature. - Scaling products without other prior knowledge.	- The scale domain is not fully understood.	[78] [47] [80]
